# Unexpected diversity of cnidarian integrins: expression during coral gastrulation

**DOI:** 10.1186/1471-2148-8-136

**Published:** 2008-05-09

**Authors:** Brent A Knack, Akira Iguchi, Chuya Shinzato, David C Hayward, Eldon E Ball, David J Miller

**Affiliations:** 1 ARC Centre of Excellence for Coral Reef Studies, James Cook University, Townsville, Queensland, 4811, Australia; 2 Centre for the Molecular Genetics of Development and Research School of Biological Sciences, Australian National University, P. O. Box 475 Canberra, ACT, 2601, Australia

## Abstract

**Background:**

Adhesion mediated through the integrin family of cell surface receptors is central to early development throughout the Metazoa, playing key roles in cell-extra cellular matrix adhesion and modulation of cadherin activity during the convergence and extension movements of gastrulation. It has been suggested that *Caenorhabditis elegans*, which has a single β and two α integrins, might reflect the ancestral integrin complement. Investigation of the integrin repertoire of anthozoan cnidarians such as the coral *Acropora millepora *is required to test this hypothesis and may provide insights into the original roles of these molecules.

**Results:**

Two novel integrins were identified in *Acropora*. AmItgα1 shows features characteristic of α integrins lacking an I-domain, but phylogenetic analysis gives no clear indication of its likely binding specificity. AmItgβ2 lacks consensus cysteine residues at positions 8 and 9, but is otherwise a typical β integrin. In situ hybridization revealed that AmItgα1, AmItgβ1, and AmItgβ2 are expressed in the presumptive endoderm during gastrulation. A second anthozoan, the sea anemone *Nematostella vectensis*, has at least four β integrins, two resembling AmItgβ1 and two like AmItgβ2, and at least three α integrins, based on its genomic sequence.

**Conclusion:**

In two respects, the cnidarian data do not fit expectations. First, the cnidarian integrin repertoire is more complex than predicted: at least two βs in *Acropora*, and at least three αs and four βs in *Nematostella*. Second, whereas the bilaterian αs resolve into well-supported groups corresponding to those specific for RGD-containing or laminin-type ligands, the known cnidarian αs are distinct from these. During early development in *Acropora*, the expression patterns of the three known integrins parallel those of amphibian and echinoderm integrins.

## Background

Integrins are a large family of cell surface transmembrane receptors known only from metazoans, which function in intracellular signalling as well as cell-cell and cell-extracellular matrix (ECM) adhesion [1]. As the main mediators of cell-ECM interactions they are key players in early development [2] functioning in gastrulation by rapid modulation of their own adhesion between low and high affinity states and by modulating the activities of adhesion molecules (e.g. cadherins) in cell layers undergoing convergence and extension [3].

Integrins function as αβ heterodimers with several subunits of each type being present in most animals. Analyses of the whole genome sequence of *Caenorhabditis elegans *[4,5] indicate that it has a single β subunit of the β1 type that is capable of associating with two α subunits, which confer specificity for either laminin- or RGD-containing ligands, and it has been suggested that this may reflect the ancestral state. *Drosophila melanogaster *has five α and two β subunits [5] and mammals, eighteen α and eight β integrin subunits [6]. In each case, however, integrin subunits above and beyond likely orthologs of the two αs and one β of *Caenorhabditis *are clearly lineage-specific. "Lower" animals are of particular significance in terms of understanding the ancestral state, but have not been extensively studied. Both α and β integrin subunits have been identified in sponges [7-9], and cnidarians [7,10,11], but the extent of integrin diversity and the range of functions of these molecules in "lower" animals are unknown.

Anthozoan cnidarians such as the coral *Acropora millepora *and the sea anemone *Nematostella vectensis *appear to have retained much of the genetic complexity of the metazoan common ancestor [12,13]; hence these animals are likely to be highly informative with respect to the ancestral integrin complement and may provide insights into the original roles of these molecules. Known cnidarian integrins include a β integrin from *Acropora millepora *[7] and single α and β subunits (IntA and IntB) from the hydrozoan jellyfish *Podocoryne carnea *[11]. Here we report the characterisation of novel β and α integrins from *Acropora*. The known *Acropora *integrins are expressed during gastrulation in patterns like those seen at the corresponding stages of echinoderm and amphibian development. However, we know from morphological observation [14,15] that *Acropora *gastrulation is not a simple epithelial to mesenchymal transition in which the expressing cells lose their adhesivity and invaginate. Instead, in *Acropora *it is clear that changing cell shape also plays a major role[14]. Two further implications of this work are that the cnidarian integrin complement is significantly more complex than was predicted, and that functional diversification of α integrins may have occurred independently in Cnidaria and Bilateria.

## Results

### Identification of novel integrins

Three unigenes encoding integrin subunits were identified during an ongoing EST analysis of *Acropora millepora *[13,16]. One of these corresponds to the previously known *Acropora *integrin β Cn1 [7]; to simplify comparative analyses, this integrin is henceforth referred to as AmItgβ1. Complete sequences were determined for cDNA clones corresponding to the other two integrin unigenes; comparative analyses indicated that an EST clone corresponding to a second β subunit (AmItgβ2) encodes a full length protein of 771 amino acids, whilst an α integrin EST clone lacked the 5' end of the open reading frame. To complete the 1021 amino acid integrin α coding sequence, overlapping clones were isolated from a cDNA library, enabling the determination of the complete open reading frame for a molecule designated AmItgα1. These sequences have been submitted to GenBank under the following accession numbers: EU239371 (AmItgα1) and EU239372 (AmItgβ2).

### AmItgβ2 is a possible coral ortholog of a known jellyfish integrin β

**Figure 1:**
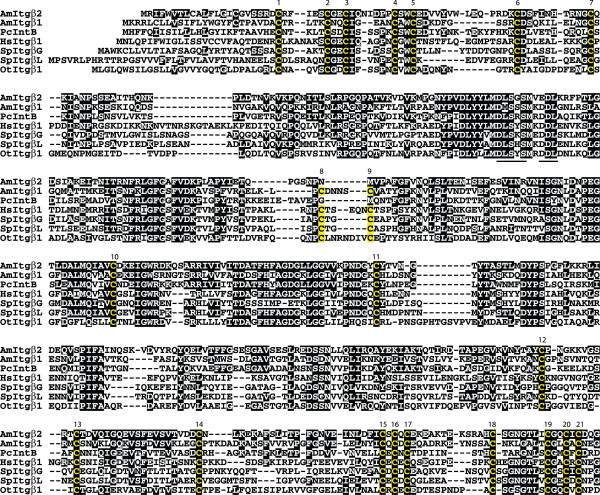
**β integrin alignments (amino terminal end of the molecules)**. Amino acid sequence of AmItgβ2 aligned with representative β integrin sequences. Atypical absence of cysteines (yellow, numbered) from positions 8 and 9 suggests orthology between AmItgβ2 and *Podocoryne *IntB (PcIntB). Structural features including the MIDAS motif (DLSXS, underlined), transmembrane region (long wavy line), membrane proximal motif (short wavy line), and two NPxY/F motifs (double underline) are conserved. The ADMIDAS motif (DDL, underlined) is changed to EDL in AmItgβ2. An arrow indicates the position where a deletion was made in the sponge sequence (OtItgβ1) to facilitate alignment. Abbreviations and database accession numbers for sequences used in the alignment are: *Acropora *AmItgβ2 (AmItgβ2; EU239372); *Podocoryne *IntB (PcIntB; AAG25994); *Acropora *AmItgβ1 (AmItgβ1; AAB66910); Human β1 (HsItgβ1; P05556); *Strongylocentrotus *βG (Urchin SpItgβG; AAB39739); *Strongylocentrotus *βL (SpItgβL; AAC28382); *Ophlitaspongia *βPo1 (Sponge OtItgβ1; AAB66911).

**Figure 2:**
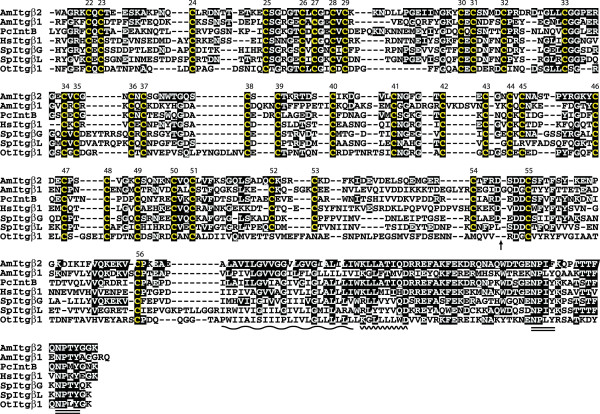
**β integrin alignments (carboxy terminal end of the molecules)**. See legend for Fig. 1.

Database comparisons identified AmItgβ2 as a possible ortholog of integrin β (IntB; Q9GSF3) from *Podocoryne *[11]. Previously Reber-Muller et al. [11] suggested that *Podocoryne *IntB was orthologous with AmItgβ1; however, the former not only has higher overall sequence identity with AmItgβ2 (44% amino acid identity compared to <40%), but also shows the same atypical pattern of cysteine residues (Fig. 1, 2) Whereas the β integrin extracellular domain characteristically contains 56 cysteine residues arranged in a specific pattern [7], in both *Podocoryne *IntB [11] and AmItgβ2, cysteine residues at positions 8 and 9 in the canonical structure are absent as occurs in vertebrate β4-type integrins. In terms of most other structural features, however, both AmItgβ2 and *Podocoryne *IntB are typical integrin βs – the MIDAS domain, cysteine-rich stalk and transmembrane region are all clearly present. In both cases, the DxSxS motif of the MIDAS cation-binding domain [17] is completely conserved, whereas the DDL motif of the ADMIDAS is changed to EDL in AmItgβ2 (Fig. 1). The cytoplasmic domains contain the conserved membrane proximal sequence KLLxxxxD and two NPxY/F motifs (NPIF and NPTY in AmItgβ2, NPIY and NPMY in IntB). Whereas the degree of similarity between these *Acropora *and *Podocoryne *sequences implies that these might be orthologs, one complicating factor is that the *Nematostella *genome appears to encode two integrins of the AmItgβ2 type (see below). Hence orthology relationships will only be clear when more complete datasets are available for *Acropora *and *Podocoryne*.

### AmItgα1 is a cnidarian integrin resembling the vertebrate α4/9-type

**Figure 3:**
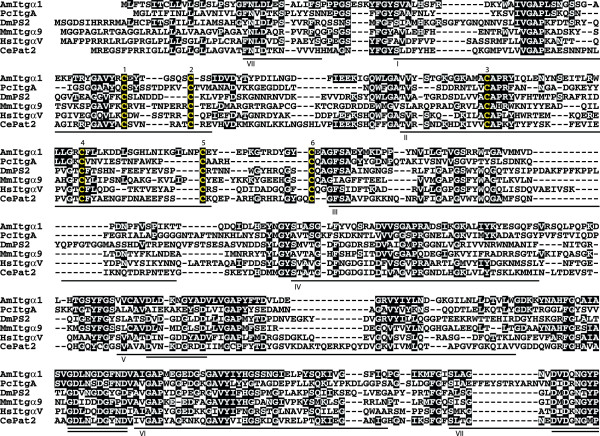
**α integrin alignments (amino terminal end of the molecules)**. The major structural features of alpha integrins lacking an alpha-A domain are conserved in AmItgα1 including seven FG-GAP repeats (underlined, roman numerals), three DxD/NxD/NxxxD cation binding sites (double underline), the transmembrane region (long wavy line) and the cytosolic membrane proximal domain (short wavy line). The position of a putative fourth cation binding site in the *Podocoryne *sequence is indicated in red. Arrows mark the positions where regions that could not be unambiguously aligned were removed from the *Drosophila *(DmPS2; 219 residues), *Caenorhabditis *(CePat2; 132 residues) and human (HsItgαV; 6 residues) sequences. Abbreviations and database accession numbers for sequences used in the alignment are: *Acropora *AmItgα1 (AmItgα1; EU239371); *Podocoryne *IntA (PcIntA; AAG25993); *Drosophila *αPS2 (DmPS2; P12080); Mouse α9 (MmItgα9; NP_598482); Human αV (HsItgαV; P06756); *Caenorhabditis *αPat2 (CePat2; P34446).

**Figure 4:**
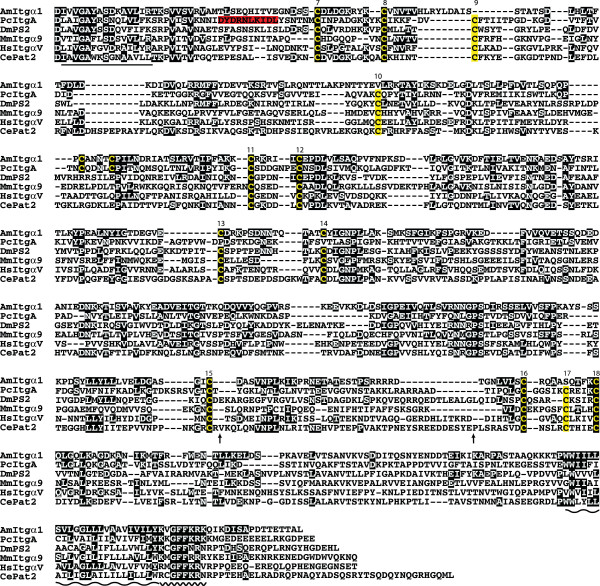
**α integrin alignments (carboxy terminal end of the molecules)**. See legend for Fig. 3.

Comparative analyses indicate that AmItgα1 shares some characteristics with those integrin α subunits that lack an α-A (I) domain. Database comparisons revealed that AmItgα1 is most similar to mouse integrin α9 (MmItgα9; Q91YD5; 28% identity and 48% similarity). Whereas the *Acropora *and *Podocoryne *β integrins that are possible orthologs (AmItgβ2 and PcIntB) are 44% identical, the α subunits AmItgα1 and *Podocoryne *IntA have a much lower amino acid identity (27%), and there are several differences between these that are likely to have functional and/or structural significance. Both proteins are typical in terms of the presence of FG-GAP repeats, transmembrane regions, membrane proximal KxGFFKR motifs and extracellular cleavage sites fitting the RxK/RR consensus (Fig. 3, 4). However, whereas AmItgα1 is typical in having three cation binding motifs (DxD/NxD/NxxxD; [18]) within FG-GAP repeats V, VI, and VII, the three cation binding sites in *Podocoryne *IntA are in FG-GAP repeats VI, VII and immediately C-terminal of repeat VII. Both proteins are atypical in terms of the positions of cysteine residues relative to the consensus; AmItgα1 is missing Cys residues at positions 9, 10 and 17, whereas IntB is missing Cys residues at positions 13 and 14, but both proteins have a novel Cys pair between consensus positions 10 and 11. A corresponding extra pair of Cys residues is also present at the same position in both the *Nematostella *α integrin predicted from the genome sequence (NvItgα1; see below) and the atypical *Drosophila *integrin PS3.

### Phylogenetic analyses of the novel integrin sequences

To better understand relationships between the *Acropora *sequences and the major integrin types of higher animals, maximum likelihood (ML) phylogenetic analyses were undertaken using MolPhy version 2.3 [19]. Integrin phylogenetics is complicated by high levels of primary sequence divergence and homoplasy, leading to difficulties in unambiguous alignment of sequences. The analyses presented here are therefore based on extensively edited alignments. Sequences were aligned using ClustalW via the EBI website, manually edited using JalView, and used for phylogenetic analyses. In the case of the integrin α alignment, the output from ClustalW consisted of 2383 positions and was manually edited to 1091 positions (20 sequences). The corresponding figures for the integrin β alignments were 2159 positions prior to editing and 991 after editing (24 sequences).

**Figure 5:**
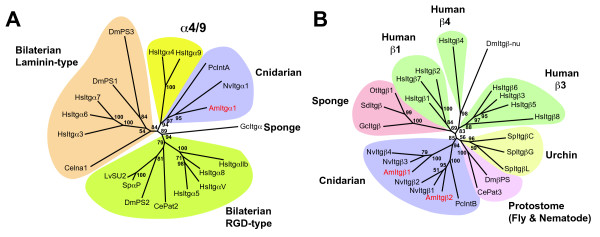
**Maximum likelihood phylogenetic analysis of representative α and β integrin proteins**. Numbers at branch points indicate the percentage of 1000 bootstrap replicates supporting the topology shown (using MolPhy version 2.3; see [14]). (A) α integrins. Whereas integrins from Bilateria group in a ligand specific manner, consistent with previous phylogenies, the cnidarian sequences form an independent clade, reflecting their early divergence. These groupings suggest that functional divergence of α integrins had already occurred in the Urbilateria. Sequences aligned, abbreviations, and accession numbers are: *Lytechinus *SU2 (LvSU2; AAC23572); *Strongylocentrotus *αP (SpαP; AAD55724); *Drosophila *αPS2 (DmPS2; P12080); Human α5 (HsItgα5; P08648); Human αV (HsItgαV; P06756); Human α8 (HsItgα8; P53708); Human αIIb (HsItgαllb; P08514); Human α6 (HsItgα6; P23229); Human α7 (HsItgα7; Q13683); Human α3 (HsItgα3; P26006); *Drosophila *αPS3 (DmPS3; O44386); *Acropora *AmItgα1 (AmItgα1; EU239371); Human α4 (HsItgα4; P13612); Human α9 (HsItgα9; Q13797); *Nematostella *NvItgα1 (NvItgα1; XP_001641435); *Caenorhabditis *αPat2 (CePat2; P34446); *Drosophila *αPS1 (DmPS1; Q24247); *Podocoryne *IntA (PcIntA; AAG25993); *Caenorhabditis *αIna1 (CeIna1; Q03600); *Geodia *α (GcItgα; CAA65943). (B) β integrins. Major clades resolved here are consistent with previous phylogenies. The position of sequences within the cnidarian clade is consistent with orthology between *Podocoryne *IntB (PcIntB) and AmItgβ2, and groups two *Nematostella *βs with each *Acropora *β. Unlike the α integrins, the β integrins appear to have diverged independently in several bilaterian lineages. Sequences aligned, abbreviations, and accession numbers are: Human β3 (HsItgβ3; P05106); Human β5 (HsItgβ5; P18084); Human β6 (HsItgβ6; P18564); Human β2 (HsItgβ2; P05107); Human β7 (HsItgβ7; P26010); Human β1 (HsItgβ1; P05556); *Strongylocentrotus *βG (Urchin SpItgβG; AAB39739); *Strongylocentrotus *βL (SpItgβL; AAC28382); *Strongylocentrotus *βC (SpItgβC; AAB39740); *Drosophila *βPS (DmβPS P11584); *Caenorhabditis *βPat3 (CePat3; Q27874); *Acropora *AmItgβ2 (AmItgβ2; EU239372); *Nematostella *β1 (NvItgβ1; XP_001641468); *Nematostella *β2 (NvItgβ2; XP_001627336); *Podocoryne *IntB (PcIntB; AAG25994); *Acropora *AmItgβ1(AmItgβ1; AAB66910); *Nematostella *β3 (NvItgβ3; XP_001637894); *Nematostella *β4 (NvItgβ4; XP_001621822); *Ophlitaspongia *βPo1 (Sponge OtItgβ1; AAB66911); *Suberites *β (Sponge SdItgβ; CAB38100); *Geodia *β (Sponge GcItgβ; CAA77071); Human β4 (HsItgβ4; P16144); *Drosophila *β-nu (Dmβ-nu; Q27591); Human β8 (HsItgβ8; P26012).

The ML phylogenetic analyses of integrin α sequences (Fig. 5A) are broadly consistent with previous studies; the resolution of bilaterian sequences into two major clades corresponding to the major ligand classes RGD (PS2) and laminin (PS1) is strongly supported. The fact that these two clades each contain protostome (fly, worm) and deuterostome (human, sea urchin) sequences indicates that the functional divergence of α integrins had already occurred in Urbilateria – the common ancestor of bilateral (higher) animals. However, the ML phylogenetic analyses give no clear indication of the likely ligand specificity of the known cnidarian α integrins. The cnidarian (*Podocoryne*, *Acropora *and *Nematostella*) α integrins group together with high bootstrap support (albeit on long braches which reflects their divergence), the sister group of this cnidarian integrin α clade being the α4/9 type integrins (Fig. 5A).

ML analyses of the integrin β sequences (Fig. 5B) confirmed the possible orthology of *Podocoryne *IntB with the novel *Acropora *β sequence (AmItgβ2) reported here. Preliminary surveys of the genome sequence of *Nematostella *suggest the presence of at least four β integrins, and gene models (the gene as predicted in the genome assembly, including the open reading frame, introns and untranslated regions) of these were sufficiently complete for them to be included in phylogenetic analyses (Fig. 5B) which group the *Nematostella *β integrins with the *Acropora *subunits – two with AmItgβ1 and two with AmItgβ2. It remains to be seen whether each member of these pairs of *Nematostella *genes has an *Acropora *ortholog. As in the integrin α phylogeny, the sponge sequences were relatively distant to those from the Cnidaria. Again, the analyses were broadly consistent with previous studies [20-22], resolving the vertebrate sequences into three clades known as β1 (integrin β1/2/7), β3 (integrin β3/5/6/8) and β4 in the Hughes [20] phylogeny. Unlike the α integrins, there is no evidence for divergence of β subunits prior to the protostome/deuterostome split. Rather, it appears more likely that β integrins have diverged independently in several bilaterian lineages.

### Expression of integrins during coral gastrulation

**Figure 6:**
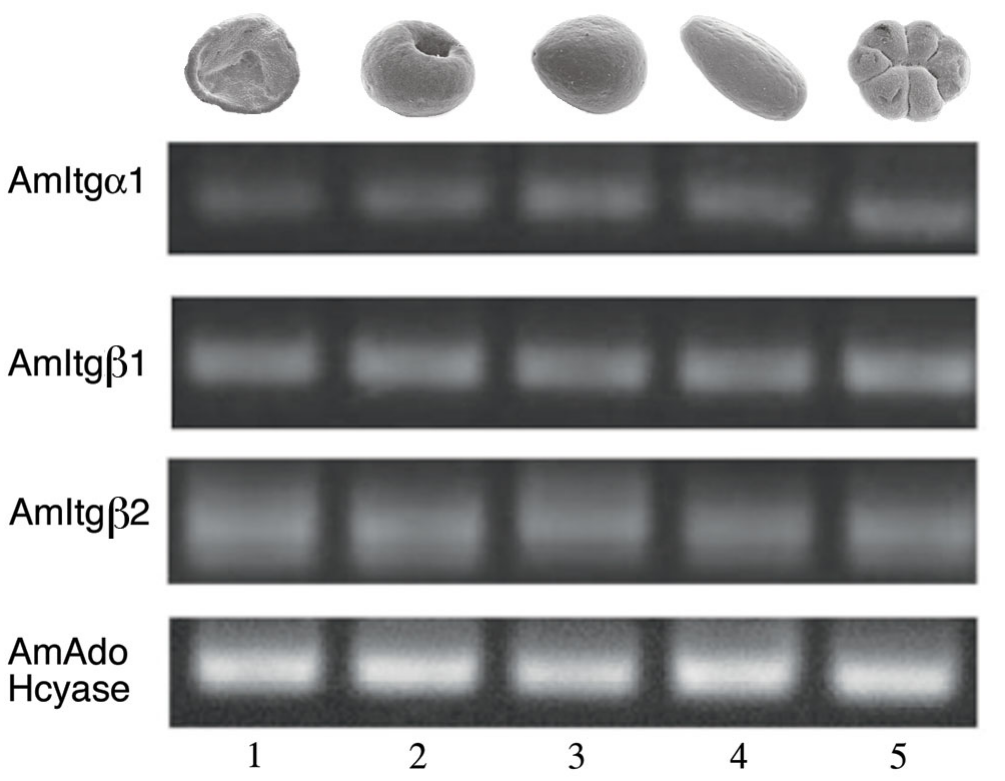
**Reverse transcriptase PCR analysis of integrin expression during *Acropora *development**. Time points: 1-Prawn Chip, 2-Gastrula, 3-Pear, 4-Planula, 5-Settlement. All three *Acropora *integrin subunits show constant levels of expression throughout development.

**Figure 7:**
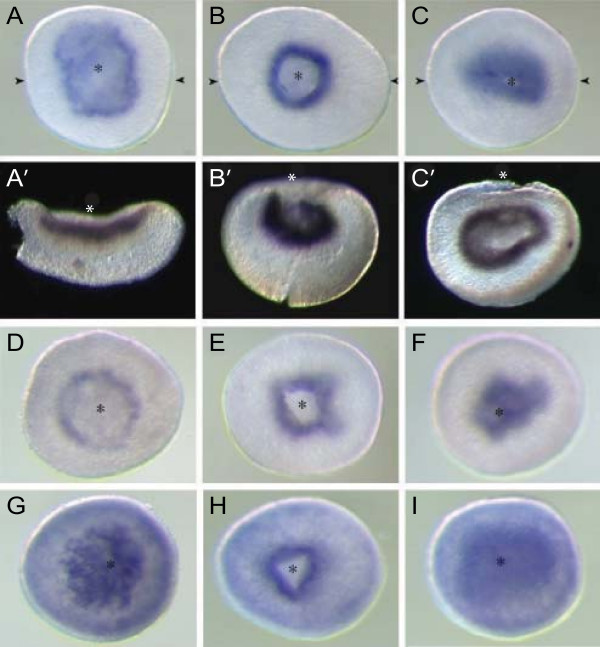
**Comparison of AmItgα1, AmItgβ1 and AmItgβ2 mRNA distribution patterns during gastrulation in *Acropora***. At the prawn chip stage, the AmItgα1 and AmItgβ1 mRNAs are clearly restricted to one side of the flattened cell bilayer (A, A', D). During gastrulation, these mRNAs are tightly restricted to the area of the blastopore (asterisks) lip (B, B', C, C', E, F), and throughout development remain endodermal. The distribution of AmItgβ2 mRNA (G, H, I) is broadly similar to that of AmItgα1, but is less tightly restricted, as indicated by weak general staining. Arrow heads in A, B, and C indicate the plane of the sections shown as A', B' and C'.

RT-PCR analysis demonstrates that mRNAs encoding each of the coral integrins (AmItgα1, AmItgβ1 and AmItgβ2) are present in eggs (Knack et al., unpublished data; [23]) and early developmental stages (Fig. 6) at relatively uniform levels. No specific pattern could be detected via in situ hybridization prior to the prawn chip stage however, implying that the maternal mRNA is uniformly distributed until this time. In the early development of *Acropora*, a clear and specific in situ hybridization pattern for AmItgα1 is first seen in late prawn chip stage embryos (early gastrula) (Fig. 7A), the mRNA being localized in a characteristic pattern on the concave side of the flattened cell bilayer corresponding to Fig. 4b in Hayward et al. [14]. Expression is maintained in these presumptive endodermal cells as the concavity deepens and they are internalized. Staining remains strong in the presumptive endoderm until blastopore closure is complete (Fig. 7C), after which only weak endodermal staining of AmItgα1 is observed.

The early expression patterns of both AmItgβ1 and AmItgβ2 (Fig. 7) were broadly similar to that of AmItgα1, but with the following differences. First, it was only possible to visualize the localization of transcripts corresponding to the β integrins at slightly later stages of development. Second, whereas AmItgα1 and AmItgβ1 transcripts were tightly restricted at the area of the blastopore lip in early gastrulae (Fig 7B and 7E), AmItgβ2 was also expressed more generally (Fig 7G–I).

## Discussion

Whereas Reber-Muller et al. [11] hypothesized the presence of only single α and β integrin subunits in cnidarians, the integrins identified to date in *Acropora *are likely to be only a subset of those present. Preliminary surveys of the genome of *Nematostella *imply that at least four β integrins (two resembling AmItgβ1 and two more similar to AmItgβ2), and at least three distinct α types are present (data not shown). However, gene models for only one α integrin were sufficiently complete to allow its inclusion in the phylogenetic analyses shown as Fig 5A. The integrin repertoire of this morphologically simple animal is therefore considerably more complex than that of *Caenorhabditis *(one β and two αs), which has often been assumed to reflect the ancestral metazoan state.

Due to structural constraints on integrin proteins during evolution, phylogenetic analyses are likely to be complicated by homoplasy effects. The position of the cnidarian sequences in the α integrin phylogeny (Fig. 5A) is likely due to both the early divergence of the Cnidaria and homoplasy effects. Hence, in this case, phylogenetics is not informative as to ligand binding properties. It is likely that in cnidarians, as in higher animals, distinct α integrin types participate in binding to laminin and RGD-containing ligands, but functional analyses are required to verify this hypothesis.

Despite having a common pattern of cysteine loss, phylogenetic analyses (Fig 5B) indicate that AmItgβ2 is only distantly related to the vertebrate β4-type. Those cysteine residues (positions 8 and 9) in the consensus absent from the *Acropora *and *Podocoryne *sequences form the c8–c9 loop of the β A domain, which has been implicated in determining integrin-ligand specificity, specificity of α-β interactions, and signalling properties [24-28]. Whilst this loss has apparently occurred independently of that leading to the vertebrate β4 type, it may result in common consequences for ligand and/or α-subunit specificity.

The presence of maternal integrin mRNAs and their relatively uniform expression through development reported here for *Acropora *have precedents in *Podocoryne *[11] as well as in higher animals. Co-localization of mRNAs for AmItgβ1, AmItgβ2 and AmItgα1 suggests that either or both of the β subunits associate with the α1 subunit. There are many precedents from bilaterians for RGD-type αs associating with β1-type βs (α5β1; αVβ1; α8β1; PS2βPS). Of these, α5β1 and PS2βPS have been implicated as regulators of gastrulation in vertebrates [29,30] and *Drosophila *[31] respectively. In *Podocoryne*, IntA is assumed to associate with IntB (a possible AmItgβ2 ortholog) since they are co-expressed in a wide variety of locations over a range of life cycle stages [11], suggesting that AmItgβ2 may associate with the AmItgα1 subunit.

Although integrins clearly play important roles in gastrulation in several animal groups, including vertebrates [29], *Drosophila *[31], and sea urchins [32], the interactions that have been demonstrated are heterogeneous with respect to both the ligands and types of integrins involved. The expression patterns of α and β integrin genes observed in the coral are reminiscent of expression during both amphibian and sea urchin gastrulation. Modulation of integrin adhesion to the RGD domain of fibronectin plays a central role during *Xenopus *gastrulation [33], and in the sea urchin *Lytechinus *changes in laminin adhesion mediated by the epithelial α integrin α SU2 are likewise important [34]. In both the sea urchin and *Xenopus*, changes in integrin-mediated cell adhesion during gastrulation occur independently of transcription and translation. In *Acropora*, integrin mRNAs are present in eggs [23] and the extent to which those visualized in the presumptive endoderm reflect zygotic transcription is unknown.

The simplest interpretation of the patterns of integrin expression is that they reflect increases in cell adhesion in the presumptive endoderm. Expression on the concave side of the "fat prawn chip" stage embryo, which is essentially a flat bilayer of cells (for a description of gastrulation in *Acropora*, see [14]), suggests that integrin-based adhesion may constrain the presumptive endoderm whilst the cells of the presumptive ectoderm move upward and inward around them. Whilst the cells that will end up inside the embryo may adhere to each other more tightly than they do to the putative ectoderm, as is consistent with many forms of gastrulation, the inferred increased adhesion in the putative endoderm is not consistent with a typical epithelial to mesenchymal transition.

The early expression patterns of the α and β integrins in *Acropora *are very similar to those of two transcription factors, *snailA *[15] and *otxB *[35]. Snail genes have central and conserved roles in gastrulation in *Drosophila *[36] and vertebrates [37] and members of the broader class of related genes are regulators of other epithelial to mesenchymal transitions (EMTs). Whilst the best understood means by which snail genes regulate cell adhesion is by acting as repressors of E-cadherin expression [36,38,39], in human epidermal keratinocytes the snail-related gene Slug (Snail2) is a repressor of α3, β1 and β4 integrin expression, leading to decreased cell-adhesion to fibronectin and laminin 5 [40]. Across the Bilateria, Otx genes are conserved anterior markers [41] and, whilst most Otx genes are expressed in the nervous system, evidence from a diverse range of metazoans suggests an ancient role as regulators of cell adhesion (eg. [42,43]). In *Hydra*, high levels of CnOtx expression correspond to regions where cells are undergoing rearrangements or movement [44]. Both *snailA *and *otxB *are thus candidate regulators of integrin expression in *Acropora*. Given the similarity of AmItgα1 to diverged RGD-type mammalian integrins, it will be of particular interest to examine the expression of ECM proteins containing fibronectin type III domains during cnidarian gastrulation in parallel with adhesion studies. Candidates identified in *Nematostella *include predicted proteins similar to vertebrate usherin and titin, and a likely homolog of *Drosophila *sidekick.

## Conclusion

Whilst one might expect morphologically simple metazoans to have a correspondingly basic integrin complement, comprising perhaps just two αs and a single β subunit (as in *Caenorhabditis elegans*), the repertoire of these molecules in anthozoan cnidarians is considerably more complex. In the case of cnidarian α integrins, ligand specificity cannot be predicted by phylogenetic analysis, suggesting the possibility that specificity mechanisms arose independently in Cnidaria and Bilateria. During early development in *Acropora*, some of these adhesion/signalling molecules are expressed in patterns which parallel those of their amphibian and echinoderm counterparts, and which are inconsistent with gastrulation being a simple epithelial to mesenchymal transition.

## Methods

### Sample collection and RNA extraction

Developmentally staged *Acropora millepora *embryos were collected during annual spawning events. Embryos were staged based on Ball et al. (2002). Total RNA was extracted using RNAWIZ (Ambion) according to the manufacturer's protocol.

### RT-PCR analysis

RNA was treated with DNase (Fermentas) to remove contaminating genomic DNA. Single stranded cDNA was synthesised using the First-strand cDNA Synthesis Kit (Amersham Biosciences, Piscataway, NJ) using 1 μg of total RNA. One μl of this product was used as a polymerase chain reaction (PCR) template. For AmItgα1, primers AmItga1RTF (5'-GCCAATGAAACAGCTACG-3') and AmItga1RTR (5'-TTGTCTCCAGCCTTCAAC-3') were used to amplify a 130 bp product. For AmItgβ2, primers AmItgb2RTF (5'-TGGGCATTTGTGGTGTGAG-3') and AmItgb2RTR (5'-GCTTGTTCTGATGAGTGATGG-3') were used to amplify a 219 bp product. For AmItgβ1 (Brower et al. 1997), primers IB1RTF (5'-CTTGTGTTGCCACTTATGGCTT-3') and IB1RTR (5'-CTGCTACTTGCATTAACGCATC-3') were used to amplify a 144 bp product. The PCR protocol was 1 min at 94°C, then 40 cycles of 0.5 min at 94°C (denaturation), 0.5 min at 50°C (annealing), 2 min at 72°C (extension), followed by an additional extension for 2 min at 72°C.) As a control, primers ADH-F (5'-AAGAAGACAAACATCAAGCCTCA-3') and ADH-R (5'-CACATCCAAGGTTCACAAGACG-3') were used to amplify a portion of coral AdoHcyase (S-adenosyl-L-homocysteine hydrolase) cDNA (unpublished data).

### Whole mount in situ hybridization

The basic procedures for fixation and hybridization with coral embryos were carried out as described [45]. Photographs were captured directly with a Spot digital camera. Digitised images were processed with Adobe Photoshop.

## Authors' contributions

BAK was responsible for conducting DNA sequencing and RT-PCR analysis, and participated in both the in situ hybridisation experiments and preparing the manuscript. AI collected and prepared coral developmental stages, assisted in DNA sequencing and, with CS, carried out in situ hybridisation experiments and photographed the results. DCH was responsible for the initial identification of clones, assisted in sequence alignment and critically reviewed both the data and the manuscript. DJM conducted the phylogenetic analyses, and he and EEB drafted and reviewed the manuscript.

## References

[B1] Hynes RO Integrins: bidirectional, allosteric signaling machines Cell 2002 110 673 687 10.1016/S0092-8674(02)00971-6 12297042

[B2] De Arcangelis A Georges-Labouesse E Integrin and ECM functions: roles in vertebrate development Trends Genet 2000 16 389 395 10.1016/S0168-9525(00)02074-6 10973067

[B3] Marsden M DeSimone DW Integrin-ECM interactions regulate cadherin-dependent cell adhesion and are required for convergent extension in Xenopus Curr Biol 2003 13 1182 1191 10.1016/S0960-9822(03)00433-0 12867028

[B4] consortium C Genome sequence of the nematode C. elegans: a platform for investigating biology Science 1998 282 2012 2018 10.1126/science.282.5396.2012 9851916

[B5] Adams MD Celniker SE Holt RA Evans CA Gocayne JD Amanatides PG Scherer SE Li PW Hoskins RA Galle RF George RA Lewis SE Richards S Ashburner M Henderson SN Sutton GG Wortman JR Yandell MD Zhang Q Chen LX Brandon RC Rogers YH Blazej RG Champe M Pfeiffer BD Wan KH Doyle C Baxter EG Helt G Nelson CR Gabor GL Abril JF Agbayani A An HJ Andrews-Pfannkoch C Baldwin D Ballew RM Basu A Baxendale J Bayraktaroglu L Beasley EM Beeson KY Benos PV Berman BP Bhandari D Bolshakov S Borkova D Botchan MR Bouck J Brokstein P Brottier P Burtis KC Busam DA Butler H Cadieu E Center A Chandra I Cherry JM Cawley S Dahlke C Davenport LB Davies P de Pablos B Delcher A Deng Z Mays AD Dew I Dietz SM Dodson K Doup LE Downes M Dugan-Rocha S Dunkov BC Dunn P Durbin KJ Evangelista CC Ferraz C Ferriera S Fleischmann W Fosler C Gabrielian AE Garg NS Gelbart WM Glasser K Glodek A Gong F Gorrell JH Gu Z Guan P Harris M Harris NL Harvey D Heiman TJ Hernandez JR Houck J Hostin D Houston KA Howland TJ Wei MH Ibegwam C Jalali M Kalush F Karpen GH Ke Z Kennison JA Ketchum KA Kimmel BE Kodira CD Kraft C Kravitz S Kulp D Lai Z Lasko P Lei Y Levitsky AA Li J Li Z Liang Y Lin X Liu X Mattei B McIntosh TC McLeod MP McPherson D Merkulov G Milshina NV Mobarry C Morris J Moshrefi A Mount SM Moy M Murphy B Murphy L Muzny DM Nelson DL Nelson DR Nelson KA Nixon K Nusskern DR Pacleb JM Palazzolo M Pittman GS Pan S Pollard J Puri V Reese MG Reinert K Remington K Saunders RD Scheeler F Shen H Shue BC Siden-Kiamos I Simpson M Skupski MP Smith T Spier E Spradling AC Stapleton M Strong R Sun E Svirskas R Tector C Turner R Venter E Wang AH Wang X Wang ZY Wassarman DA Weinstock GM Weissenbach J Williams SM WoodageT Worley KC Wu D Yang S Yao QA Ye J Yeh RF Zaveri JS Zhan M Zhang G Zhao Q Zheng L Zheng XH Zhong FN Zhong W Zhou X Zhu S Zhu X Smith HO Gibbs RA Myers EW Rubin GM Venter JC The genome sequence of Drosophila melanogaster Science 2000 287 2185 2195 10.1126/science.287.5461.2185 10731132

[B6] Whittaker CA Hynes RO Distribution and evolution of von Willebrand/integrin A domains: widely dispersed domains with roles in cell adhesion and elsewhere Mol Biol Cell 2002 13 3369 3387 10.1091/mbc.E02-05-0259 12388743 PMC129952

[B7] Brower DL Brower SM Hayward DC Ball EE Molecular evolution of integrins: genes encoding integrin beta subunits from a coral and a sponge Proc Natl Acad Sci U S A 1997 94 9182 9187 10.1073/pnas.94.17.9182 9256456 PMC23098

[B8] Pancer Z Kruse M Muller I Muller WE On the origin of Metazoan adhesion receptors: cloning of integrin alpha subunit from the sponge Geodia cydonium Mol Biol Evol 1997 14 391 398 9100369 10.1093/oxfordjournals.molbev.a025775

[B9] Wimmer W Perovic S Kruse M Schroder HC Krasko A Batel R Muller WE Origin of the integrin-mediated signal transduction. Functional studies with cell cultures from the sponge Suberites domuncula Eur J Biochem 1999 260 156 165 10.1046/j.1432-1327.1999.00146.x 10091595

[B10] Muller WE Origin of metazoan adhesion molecules and adhesion receptors as deduced from cDNA analyses in the marine sponge Geodia cydonium: a review Cell Tissue Res 1997 289 383 395 10.1007/s004410050885 9232818

[B11] Reber-Muller S Studer R Muller P Yanze N Schmid V Integrin and talin in the jellyfish Podocoryne carnea Cell Biol Int 2001 25 753 769 10.1006/cbir.2000.0708 11482899

[B12] Ball EE Hayward DC Saint R Miller DJ A simple plan--cnidarians and the origins of developmental mechanisms Nat Rev Genet 2004 5 567 577 10.1038/nrg1402 15266339

[B13] Technau U Rudd S Maxwell P Gordon PM Saina M Grasso LC Hayward DC Sensen CW Saint R Holstein TW Ball EE Miller DJ Maintenance of ancestral complexity and non-metazoan genes in two basal cnidarians Trends Genet 2005 21 633 639 10.1016/j.tig.2005.09.007 16226338

[B14] Hayward DC Samuel G Pontynen PC Catmull J Saint R Miller DJ Ball EE Localized expression of a dpp/BMP2/4 ortholog in a coral embryo Proc Natl Acad Sci U S A 2002 99 8106 8111 10.1073/pnas.112021499 12048233 PMC123028

[B15] Hayward DC Miller DJ Ball EE snail expression during embryonic development of the coral Acropora: blurring the diploblast/triploblast divide? Dev Genes Evol 2004 214 257 260 10.1007/s00427-004-0398-0 15029498

[B16] Kortschak RD Samuel G Saint R Miller DJ EST analysis of the cnidarian Acropora millepora reveals extensive gene loss and rapid sequence divergence in the model invertebrates Curr Biol 2003 13 2190 2195 10.1016/j.cub.2003.11.030 14680636

[B17] Tozer EC Hughes PE Loftus JC Ligand binding and affinity modulation of integrins Biochem Cell Biol 1996 74 785 798 9164648 10.1139/o96-085

[B18] Tuckwell DS Brass A Humphries MJ Homology modelling of integrin EF-hands. Evidence for widespread use of a conserved cation-binding site Biochem J 1992 285 ( Pt 1) 325 331 1322124 10.1042/bj2850325 PMC1132784

[B19] Adachi J Hasegawa M Instability of quartet analyses of molecular sequence data by the maximum likelihood method: the Cetacea/Artiodactyla relationships Mol Phylogenet Evol 1996 6 72 76 10.1006/mpev.1996.0059 8812307

[B20] Hughes AL Evolution of the integrin alpha and beta protein families J Mol Evol 2001 52 63 72 11139295 10.1007/s002390010134

[B21] Ewan R Huxley-Jones J Mould AP Humphries MJ Robertson DL Boot-Handford RP The integrins of the urochordate Ciona intestinalis provide novel insights into the molecular evolution of the vertebrate integrin family BMC Evol Biol 2005 5 31 10.1186/1471-2148-5-31 15892888 PMC1145181

[B22] Huhtala M Heino J Casciari D de Luise A Johnson MS Integrin evolution: insights from ascidian and teleost fish genomes Matrix Biol 2005 24 83 95 10.1016/j.matbio.2005.01.003 15890260

[B23] Iguchi A Marquez LM Knack B Shinzato C van Oppen MJ Willis BL Hardie K Catmull J Miller DJ Apparent Involvement of a beta1 Type Integrin in Coral Fertilization Mar Biotechnol (NY) 2007 10.1007/s10126-007-9026-0 17694414

[B24] Bunch TA Miller SW Brower DL Analysis of the Drosophila betaPS subunit indicates that regulation of integrin activity is a primal function of the C8-C9 loop Exp Cell Res 2004 294 118 129 10.1016/j.yexcr.2003.11.002 14980507

[B25] Takagi J Kamata T Meredith J Puzon-McLaughlin W Takada Y Changing ligand specificities of alphavbeta1 and alphavbeta3 integrins by swapping a short diverse sequence of the beta subunit J Biol Chem 1997 272 19794 19800 10.1074/jbc.272.32.19794 9242639

[B26] Lin CS Chen Y Huynh T Kramer R Identification of the human alpha6 integrin gene promoter DNA Cell Biol 1997 16 929 937 9303435 10.1089/dna.1997.16.929

[B27] Takagi J DeBottis DP Erickson HP Springer TA The role of the specificity-determining loop of the integrin beta subunit I-like domain in autonomous expression, association with the alpha subunit, and ligand binding Biochemistry 2002 41 4339 4347 10.1021/bi016047u 11914080

[B28] Miao H Li S Hu YL Yuan S Zhao Y Chen BP Puzon-McLaughlin W Tarui T Shyy JY Takada Y Usami S Chien S Differential regulation of Rho GTPases by beta1 and beta3 integrins: the role of an extracellular domain of integrin in intracellular signaling J Cell Sci 2002 115 2199 2206 11973360 10.1242/jcs.115.10.2199

[B29] Davidson LA Hoffstrom BG Keller R DeSimone DW Mesendoderm extension and mantle closure in Xenopus laevis gastrulation: combined roles for integrin alpha(5)beta(1), fibronectin, and tissue geometry Dev Biol 2002 242 109 129 10.1006/dbio.2002.0537 11820810

[B30] Whittaker CA DeSimone DW Integrin alpha subunit mRNAs are differentially expressed in early Xenopus embryos Development 1993 117 1239 1249 8404528 10.1242/dev.117.4.1239

[B31] Roote CE Zusman S Functions for PS integrins in tissue adhesion, migration, and shape changes during early embryonic development in Drosophila Dev Biol 1995 169 322 336 10.1006/dbio.1995.1147 7750648

[B32] Marsden M Burke RD The betaL integrin subunit is necessary for gastrulation in sea urchin embryos Dev Biol 1998 203 134 148 10.1006/dbio.1998.9033 9806779

[B33] Ramos JW DeSimone DW Xenopus embryonic cell adhesion to fibronectin: position-specific activation of RGD/synergy site-dependent migratory behavior at gastrulation J Cell Biol 1996 134 227 240 10.1083/jcb.134.1.227 8698817 PMC2120922

[B34] Hertzler PL McClay DR alphaSU2, an epithelial integrin that binds laminin in the sea urchin embryo Dev Biol 1999 207 1 13 10.1006/dbio.1998.9165 10049560

[B35] de Jong DM Hislop NR Hayward DC Reece-Hoyes JS Pontynen PC Ball EE Miller DJ Components of both major axial patterning systems of the Bilateria are differentially expressed along the primary axis of a 'radiate' animal, the anthozoan cnidarian Acropora millepora Dev Biol 2006 298 632 643 10.1016/j.ydbio.2006.07.034 16952346

[B36] Ip YT Gridley T Cell movements during gastrulation: snail dependent and independent pathways Curr Opin Genet Dev 2002 12 423 429 10.1016/S0959-437X(02)00320-9 12100887

[B37] Carver EA Jiang R Lan Y Oram KF Gridley T The mouse snail gene encodes a key regulator of the epithelial-mesenchymal transition Mol Cell Biol 2001 21 8184 8188 10.1128/MCB.21.23.8184-8188.2001 11689706 PMC99982

[B38] Barrallo-Gimeno A Nieto MA The Snail genes as inducers of cell movement and survival: implications in development and cancer Development 2005 132 3151 3161 10.1242/dev.01907 15983400

[B39] Nieto MA The snail superfamily of zinc-finger transcription factors Nat Rev Mol Cell Biol 2002 3 155 166 10.1038/nrm757 11994736

[B40] Turner FE Broad S Khanim FL Jeanes A Talma S Hughes S Tselepis C Hotchin NA Slug regulates integrin expression and cell proliferation in human epidermal keratinocytes J Biol Chem 2006 281 21321 21331 10.1074/jbc.M509731200 16707493

[B41] Lichtneckert R Reichert H Insights into the urbilaterian brain: conserved genetic patterning mechanisms in insect and vertebrate brain development Heredity 2005 94 465 477 10.1038/sj.hdy.6800664 15770230

[B42] Bellipanni G Murakami T Doerre OG Andermann P Weinberg ES Expression of Otx homeodomain proteins induces cell aggregation in developing zebrafish embryos Dev Biol 2000 223 339 353 10.1006/dbio.2000.9771 10882520

[B43] Perea-Gomez A Lawson KA Rhinn M Zakin L Brulet P Mazan S Ang SL Otx2 is required for visceral endoderm movement and for the restriction of posterior signals in the epiblast of the mouse embryo Development 2001 128 753 765 11171400 10.1242/dev.128.5.753

[B44] Smith KM Gee L Blitz IL Bode HR CnOtx, a member of the Otx gene family, has a role in cell movement in hydra Dev Biol 1999 212 392 404 10.1006/dbio.1999.9337 10433829

[B45] Hayward DC Catmull J Reece-Hoyes JS Berghammer H Dodd H Hann SJ Miller DJ Ball EE Gene structure and larval expression of cnox-2Am from the coral Acropora millepora Dev Genes Evol 2001 211 10 19 10.1007/s004270000112 11277400

